# 158. National Cross-Sectional Study of Factors Influencing the Decision of Prescribing Penicillin as First Choice among Dentists in Japan

**DOI:** 10.1093/ofid/ofab466.360

**Published:** 2021-12-04

**Authors:** Ryuji Koizumi, Masahiro Ishikane, Yoshiki Kusama, Shinya Tsuzuki, Yusuke Asai, Yasuyuki Shimada, Chika Tanaka, Akane ono, Akihiro Kaneko, Norio Ohmagari

**Affiliations:** 1 National Center for Global health and Medicine hospital, Shinjuku, Tokyo, Japan; 2 Division of Pharmacoepidemiology, AMR Clinical Reference Center, National Center for Global Health and Medicine, Tokyo, Japan; 3 National Center for Global Health and Medicine, Shinjuku, Tokyo, Japan; 4 National Center for Global and health Medicine, chinjuku-ku, Tokyo, Japan; 5 Tokai University, Isehara, Kanagawa, Japan; 6 National Center for Global Health and Medicine Hospital, Shinjuku, Tokyo, Japan

## Abstract

**Background:**

Antimicrobial stewardship programs are needed to improve antimicrobial use among not only physicians but also dentists. This study aimed to investigate the factors influencing the decision of prescribing penicillin as first choice among dentists at clinics in Japan.

**Methods:**

We conducted a nationwide cross-sectional study of dental clinics in Japan between July and September 2020. Data on the following were collected using questionnaires: basic information, types of antimicrobials stocked, first-choice antimicrobials, and knowledge and practice of antimicrobial resistance and infectious endocarditis. Using logistic regression, odds ratios (ORs) and 95% confidence intervals (CIs) were estimated to assess the factors influencing penicillin prescription.

**Results:**

Among the 1700 participating dental clinics, 342 dental clinics responded. The median age of the study cohort was 57 (49–65) years, and there were 298 (87.1%) men. The first choice of antimicrobials was third-generation cephalosporin (169 [49.4%]), followed by penicillin (103 [30.1%]) and macrolide (19 [5.6%]). In multivariate analysis, clinics with stocked penicillin (OR = 27.30 [95% CI: 12.04–63.00]) and with more than two dentists (OR = 0.48 [95% CI: 0.24–0.92]) were associated with penicillin use as first choice.

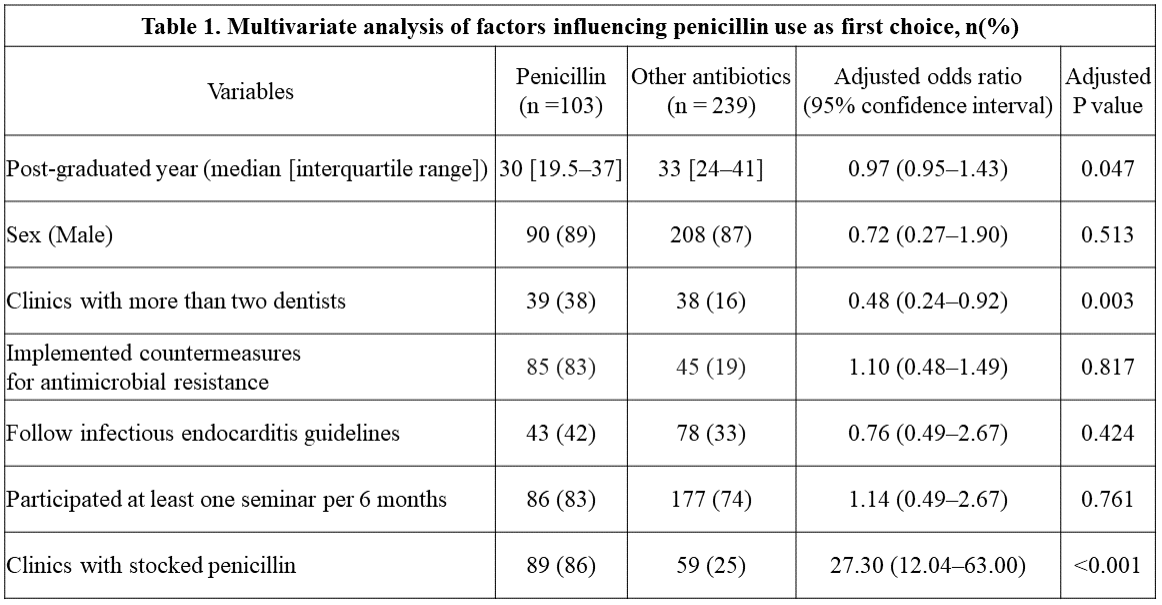

**Conclusion:**

This is the first study investigating the factors influencing the decision of prescribing penicillin as first choice among dentists in Japan. Further studies evaluating the relationships between penicillin use as first choice and stocked penicillin in the clinic and the number of working dentists are needed.

**Disclosures:**

**All Authors**: No reported disclosures

